# The different trends in the burden of neurological and mental disorders following dietary transition in China, the USA, and the world: An extension analysis for the Global Burden of Disease Study 2019

**DOI:** 10.3389/fnut.2022.957688

**Published:** 2023-01-09

**Authors:** Shan Liang, Li Wang, Xiaoli Wu, Xu Hu, Tao Wang, Feng Jin

**Affiliations:** ^1^Key Laboratory of Mental Health, Institute of Psychology, Chinese Academy of Sciences, Beijing, China; ^2^Key Laboratory of Microbial Physiological and Metabolic Engineering, Institute of Microbiology, Chinese Academy of Sciences, Beijing, China; ^3^Gut-brain Psychology Laboratory, Beijing, China; ^4^Department for the History of Science and Scientific Archaeology, University of Science and Technology of China, Hefei, Anhui, China

**Keywords:** neurological disorders, mental disorders, diet, gut microbiota, processed foods, pesticides, industrialization, fertilizer

## Abstract

**Introduction:**

The highly processed western diet is substituting the low-processed traditional diet in the last decades globally. Increasing research found that a diet with poor quality such as western diet disrupts gut microbiota and increases the susceptibility to various neurological and mental disorders, while a balanced diet regulates gut microbiota and prevents and alleviates the neurological and mental disorders. Yet, there is limited research on the association between the disease burden expanding of neurological and mental disorders with a dietary transition.

**Methods:**

We compared the disability-adjusted life-years (DALYs) trend by age for neurological and mental disorders in China, in the United States of America (USA), and across the world from 1990 to 2019, evaluated the dietary transition in the past 60 years, and analyzed the association between the burden trend of the two disorders with the changes in diet composition and food production.

**Results:**

We identified an age-related upward pattern in disease burden in China. Compared with the USA and the world, the Chinese neurological and mental disorders DALY percent was least in the generation over 75 but rapidly increased in younger generations and surpassed the USA and/or the world in the last decades. The age-related upward pattern in Chinese disease burdens had not only shown in the presence of cardiovascular diseases, neoplasms, and diabetes mellitus but also appeared in the presence of depressive disorders, Parkinson’s disease, Alzheimer’s disease and other dementias, schizophrenia, headache disorders, anxiety disorders, conduct disorders, autism spectrum disorders, and eating disorders, successively. Additionally, the upward trend was associated with the dramatic dietary transition including a reduction in dietary quality and food production sustainability, during which the younger generation is more affected than the older. Following the increase in total calorie intake, alcohol intake, ratios of animal to vegetal foods, and poultry meat to pulses, the burdens of the above diseases continuously rose. Then, following the rise of the ratios of meat to pulses, eggs to pulses, and pork to pulses, the usage of fertilizers, the farming density of pigs, and the burdens of the above disease except diabetes mellitus were also ever-increasing. Even the usage of pesticides was positively correlated with the burdens of Parkinson’s disease, schizophrenia, cardiovascular diseases, and neoplasms. Contrary to China, the corresponding burdens of the USA trended to reduce with the improvements in diet quality and food production sustainability.

**Discussion:**

Our results suggest that improving diet quality and food production sustainability might be a promising way to stop the expanding burdens of neurological and mental disorders.

## Introduction

Both neurological disorders and mental disorders are two main neuropsychiatric disorders and among the leading causes of disability globally (1, 2). Although the number of total disability-adjusted life-years (DALYs) remained almost constant from 1990 to 2019, the DALYs caused by neurological disorders and mental disorders continued to rise globally. In addition, the burdens of neurological and mental disorders are higher in high-income countries than in low-income and middle-income countries (3, 4). The progress in intervention of the above neuropsychiatric disorders did not stop or slow down the expanding of DALYs in the past decades (1, 5), and more effective prevention measures are in great need.

A growing number of studies presented the importance of gut microbiota in brain function and neurological and mental disorders (6–10). The gut microbiota and the brain communicate with each other through the microbiota–gut–brain axis (9). Improvements in brain function and/or mental health through gut microbiota regulation have been shown in many brain disorders including depressive disorders (11–13), anxiety disorders (14, 15), eating disorders (16), autism spectrum disorders (17–19), schizophrenia (20), Alzheimer’s disease (21, 22), Parkinson’s disease (22, 23), epilepsy (24, 25), and multiple sclerosis (26).

Diet is essential for brain health and specific dietary components such as tryptophan, and omega-3 polyunsaturated fatty acids (PUFAs) play a great role in normal brain function (27, 28). Diet quality is strongly linked with mental health and brain disorders (29, 30), balanced diet containing a proper combination of all kinds of nutrition could boost brain and mental health (31), while poor diet is associated with the occurrence of various brain disorders including mood disorders (32), schizophrenia (33), neurodevelopmental disorders (34), and neurodegenerative diseases (35). Furthermore, diet is the major factor modulating gut microbiota (36, 37), and microbiota regulate the intake, digestion, and utilization of nutrients (36, 38). The influences of key nutrition on the brain are closely related to gut microbiota (29, 39–41), and a growing body of research suggests the role of gut microbiota in the dietary intervention of mental and neurological disorders in the past decade (6, 29, 42–47).

A suboptimal diet is regarded as the leading risk factor for non-communicable disease burdens (48), but its DALYs are mostly calculated from the burden of cardiovascular diseases, neoplasms, and diabetes mellitus (49, 50). In addition, few studies have associated the burden of neurological disorders and mental disorders with dietary risks. Meanwhile, previous studies usually used age-standardized DALYs or prevalence to compare the burdens of different countries or regions. This measure probably underestimated the effect of differences in age structure and the influences of the human second genome (the genome of gut microbiota). Yet, in the same population, the first genome (the human genes) is relatively stable while the second genome in different generations changes following the dietary and lifestyle transitions (6, 36, 51–53). Then, following food industrialization and globalization, more and more people have been involved in industrialized/westernized diet (54–56) and been experiencing gut microbiota industrialization/westernization (51, 53, 57).

Although the whole world is going through diet industrialization, the USA and China are still typical examples of developed and developing countries, respectively, and they are at different stages of diet industrialization. The disease burden attributed to dietary risks in China is still increasing while the corresponding burden in the USA has gotten through the rise period and started to reduce (48, 50). Both countries have experienced nearly half a century of peace and developing, own large population, and are facing huge neurological and mental disease burdens. Additionally, since most Americans and Chinese have access to enough food resources, they are all desperate to improve the diet quality and prevent chronic diseases. The disease burden attributed to dietary risks was 10% higher in China than in the USA in 2017 (48), and it is time to study what caused the burden increase in China and what induced the burden decrease in the USA.

China is a rapidly developing country, which moves from a low-income country to an upper-middle income country in 22 years. China is also the most populous country (18% of the global) that has been undergoing transition from traditional diet to industrialized diet (58–61). The process of food industrialization in China is much quicker than that in the USA. Different generations have experienced different income levels and diet during the industrialization of food system and the urbanization process in the past decades, and their gut microbiota are affected by industrialized diet to different degrees. Although different Chinese generations have similar first genome, their second genome is different. It is a valuable opportunity to observe such different populations at the same time. Studying the disease changes during different generations probably helps to find the effective ways to stop the expanding disease burden in China.

The USA is the largest high-income country (4% of the world’s population), among the first countries proceeding food industrialization and probably starting industrialized diet from 1800s (62, 63). Then, the USA is also among the first countries to find the close association between western diet and common non-communicable diseases, and it has published an increasing body of research and attempts to improve the dietary quality to reduce the disease burdens (53, 55, 57, 62, 64, 65). Then, the reduction of disease burdens attributable to dietary risks in the USA indicated that some effective measures had been taken. It is necessary to study what the measures are and whether they are useful to China and other countries. Analyzing the disease differences between the USA and China in different generations not only helps to uncover the association between disease burden and diet industrialization but also contributes to find the ways to reduce the adverse effects of diet industrialization and provide the references for China, the USA, and all the world.

Since the novel severe acute respiratory syndrome coronavirus 2 (SARS-CoV-2) that causes coronavirus disease 2019 (COVID-19) can invade the brain (66), change gut microbiota (67, 68), and induce abnormal microbiota for a long time after disease resolution (69), the COVID-19 pandemic will be imposing a great impact in the epidemiology of neurological and mental disorders. A systematic review showed that the survivors of COVID-19 had higher risks of neuropsychiatric disorders such as depression, anxiety, and cognition deficits (70), and even, the general population presented a high prevalence of adverse psychiatric symptoms such as anxiety, depression, and psychological distress during the pandemic (71). At the same time, the epidemic-related prevention policies such as quarantine, restrictions on dining together, and stricter sterilization of foods have an impact on the human diet. Studies showed that there was a reduction in diet quality in several populations, especially in low-income populations and in people who were quarantined (72, 73). Thus, it is a very precious opportunity to study the relationship between the epidemiology of neurological and mental disorders with a diet transition, unaffected by the COVID-19 pandemic.

In this study, we aimed to evaluate the trend difference in neurological and mental disorders between different generations and their dietary changes in the past decades to indentify the association between the disease burden and diet transition at the national level using the data mostly from the Global Burden of Diseases, Injuries, and Risk Factors Study (GBD) 2019 and the United Nations Food and Agriculture Organization (FAO).

## Materials and methods

### Overview and definitions

In this analysis, we selected the rapidly developing China, the highly developed USA, and the world to explore the association between diet industrialization with the burdens of neurological and mental disorders. We investigated the burden trend of neurological disorders and mental disorders among people with different ages from 1990 to 2019. The neurological disorders included in GBD 2019 were headache disorders, Alzheimer’s disease and other dementias, Parkinson’s disease, idiopathic epilepsy, multiple sclerosis, motor neuron diseases, and other neurological disorders. The mental disorders included in GBD 2019 were depressive disorders, anxiety disorders, bipolar disorder, schizophrenia, autism spectrum disorders, conduct disorder, attention-deficit hyperactivity disorder, eating disorders, idiopathic developmental intellectual disability, and a residual category of other mental disorders.

The dietary risks in GBD 2019 contain 10 risks of low in certain foods and 5 risks of high in certain foods. We defined the risks of low in certain foods as a total of 10 risks as low in whole grains, low in legumes, low in vegetables, low in fruits, low in fiber, low in seafood omega-3 PUFA, low in PUFA, low in nuts and seeds, low in milk, and low in calcium. In addition, the risks of high in certain foods were defined as a total of 5 risks comprising high in sodium, high in red meat, high in processed meat, high in trans fatty acids, and high in sugar-sweetened beverages. We also included other two diet-related risks from GBD 2019: nutritional deficiencies and alcohol use. The nutritional deficiency risks included in GBD 2019 were iron deficiency, zinc deficiency, and vitamin A deficiency. In addition, the burdens induced by high body mass index (BMI) were also considered.

The dietary transition was defined as the changes in both diet composition and food production sustainability in the present study. The diet quality mainly depends on whether the nutrition combination was balanced, and poor diet quality indicators included in the study were the overconsumption of calories and alcoholic beverages, high ratio of animal foods to vegetal foods, high ratio of major meat to pulses, and high ratio of refined grains to whole grains (64). Alcohol was among the most common risks inducing diseases and a disease burden in the world (50, 74). Excessive calories, excessive refined grains but lower whole grains, and excessive animal protein but lower plant protein were all indicators of the poor quality of diet (48, 65, 75, 76). The low food production sustainability indicators came from the sustainability indicators in FAOSTAT, including the usage of fertilizer and pesticides in crop planting and the farming density of major livestock. In addition, the farming density of major livestock could indicate the usage of antimicrobics in food animals (77, 78).

Since cardiovascular diseases, neoplasms, and diabetes mellitus are greatly related to diet and are the major diseases to evaluate the burdens induced by dietary risks (49, 50), we used the three diseases as control of neurological and mental disorders.

### Data source and collection

We obtained the data on DALYs by age, country, and year from GBD 2019. The GBD approach to estimate cause-specific and risk-specific DALYs has been described in detail in another study (4).

For diet composition, we used the country availability data from FAO food balance sheets and estimated intake data from the global dietary database. For food production, we used the data from FAO sustainability and production indicator sheets, input sheets, and production sheets. The data of antimicrobial use in food animals (77, 79) and the data of excessive fertilizer (80) were obtained from previous research. We also used the body mass index (BMI) data from NCD-RisC to evaluate the body weight trend (81, 82).

In addition, Chinese data on processed foods and food processing were obtained from market investigations. The data on soft drinks, snack foods, Chinese marinating foods, instant foods, Chinese-style milk tea (a kind of attractive sweet beverage containing overdosed sugar or sweeteners and additives, which is usually prepared on site by milk tea shops and very popular in young people), baby foods, food additives, and feed additives were from qianzhan.com. The data on online food delivery, consumer data on Chinese marinating foods, and Chinese-style milk tea were obtained from iimedia.cn. The soft drink follower data came from gridsum.com, and the snack consumer data were obtained from analysys.cn. The antibiotic sale data were obtained from huaon.com.

### Data processing and analyses

The DALY percent was used in the present study in the consideration of the different population bases and population structures of different countries. The proportions of animal foods to vegetal foods, major meat to pulses, eggs to pulses, refined grains to whole grains, and different meat to total meat consumption were calculated to estimate the diet quality. The proportions of cropland and livestock at country level to the world were also calculated to estimate the difference between the USA and China. The correlation between disease burden and dietary changes was analyzed using the yearly data of China, the USA, and the world. The Spearman’s correlation was used, and then, the false discovery rate (FDR) adjustment was conducted in this analysis. Only the adjusted *p*-value of less than 0.05 was considered significant. The analyses and plotting were all proceeded in the R4.1.2.

## Results

### The different trends of neurological and mental disorders in DALY percent in different income-level countries during the past 30 years

**FIGURE 1:**
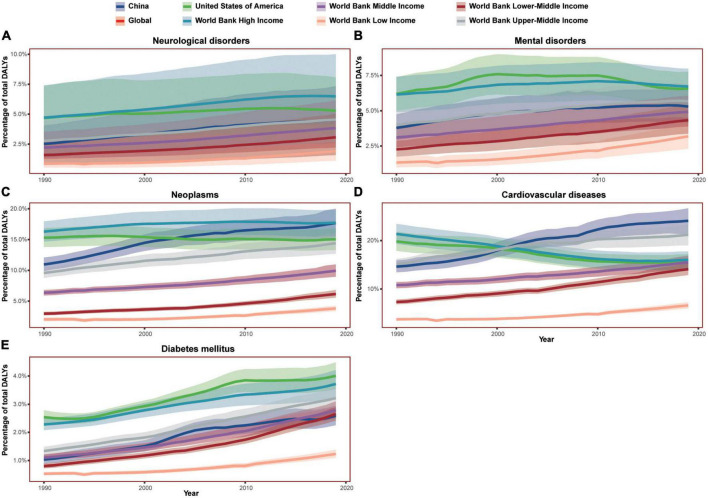
The burden trend of all-ages neurological disorders, mental disorders, and three major physiological diseases in China, the United States of America, the world, and different income countries from 1990 to 2019. **(A–E)** Showed the DALY percent of neurological disorders, mental disorders, neoplasms, cardiovascular diseases, and diabetes mellitus, respectively. The graphs of World Bank middle income overlapped with that of the world. Shaded sections indicate 95% uncertainty intervals. DALYs, disability-adjusted life-years.

In 1990, the all-ages DALY percent caused by neurological disorders, mental disorders, neoplasms, cardiovascular diseases, and diabetes mellitus in high-income countries were higher than other countries, and it seemed that the higher the income levels, the more the DALY percent (Figure 1). However, the DALY percent in countries with different income levels had shown different trends. The all-ages DALY percent caused by diabetes mellitus had been increasing from 1990 to 2019 globally. The similar trend also showed in the DALY percent caused by neurological disorders, neoplasms, and cardiovascular diseases in low-income, low-middle income, middle-income, and upper-middle income countries. However, in high-income countries, the DALY percent caused by cardiovascular diseases was decreasing from 1990 to 2019, the DALY percent caused by neoplasms peaked in 2009 and then began a slow decline, and the DALY percent caused by neurological disorders peaked in 2017. For DALYs caused by mental disorders, low-income, low-middle income, and middle-income countries presented a steady increase, while the high-income and upper-middle income countries first rose and then fell.

The USA showed a typical pattern of high-income countries, and its DALY percent reduced earlier than high-income countries in neurological disorders, neoplasms, and cardiovascular diseases. Additionally, the DALY percent of mental disorders and neurological disorders in the USA reached the peak and reduced from 2010 and 2013. Although, China was listed as an upper-middle income country after 2010, its disease burdens in neurological disorders, mental disorders, neoplasms, and cardiovascular diseases has been rapidly increasing like upper-middle income countries from 1990. The Chinese DALYs caused by neoplasms and cardiovascular diseases were even remarkably higher than upper-middle income countries. In China, the DALY percent of mental disorders peaked in 2017 while the DALY percent of neurological disorders were on a steady rise.

### The different trends of neurological and mental disorders in DALY percent in China and the USA during the past 30 years

**FIGURE 2:**
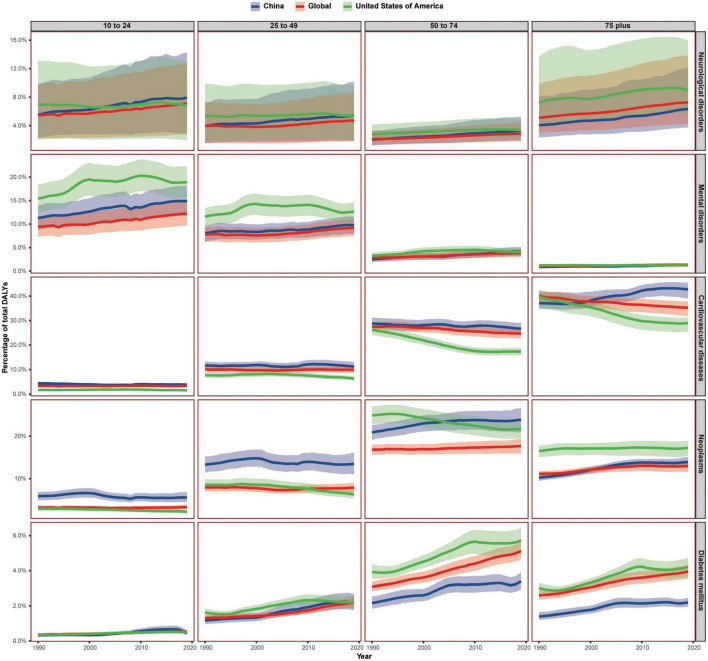
The burden trend of neurological disorders, mental disorders, and three major physiological diseases in China, the United States of America, and the world, by age group. Shaded sections indicate 95% uncertainty intervals. DALYs, disability-adjusted life-years.

Despite the all-ages Chinese DALY percent caused by mental disorders and neurological disorder were between global average and that of the USA, the corresponding DALY percent of Chinese people born in different generations showed a totally different trend (Figures 1, 2).

For mental disorders, the Chinese DALY percent of 75+ age group were lower than global average and that of the USA. The DALY percent of 50–74 age group exceeded the global average from 2008 and exceeded that of the USA from 2017. The DALY percent of 25–49, 10–24, and 0–9 age groups presented a similar increasing trend with all-ages.

For neurological disorders, the Chinese DALY percent of the 75+ age group were less than global average and that of the USA. The DALY percent of the 50–74 age group surpassed the global average from 1993. The DALY percent of the 25–49 age group surpassed the global average from 1991, and it exceeded that of the USA in 2019. The DALY percent of the 10–24 age group was also increasing rapidly, and it was higher than the global average from 1990 and surpassed that of the USA from 2003. The 0–9 age group presented a similar increasing trend with all-ages.

The age-related rapid upward trend of DALYs caused by other common diseases seemed to appear earlier in China. The all-ages cardiovascular disease DALY percent of China were higher than the global average and exceeded that of the USA in 2001. Although the DALY percent of the 0–9 age group was less than that of the USA, the DALY percent of the 75+ age group surpassed that of the USA in 1997 and surpassed that of the world from 2000, and the DALY percent of the 10–24, 25–49, and 50–74 age groups were all higher than that of the USA and the world.

The all-ages neoplasm DALY percent of China was higher than the global average and surpassed that of the USA in 2004. However, the DALY percent of the 75+ age group was less than that of the USA and exceeded the global average from 2001. The DALY percent of the 50–74 age group was more than the global average and surpassed that of the USA in 2004. The DALY percent of the 0–9 age group was more than the global average and surpassed that of the since 1996. Other age groups below 50 were all higher than that of the global average and that of the USA.

A similar upward trend of DALY percent in China appeared later for diabetes mellitus. The diabetes mellitus DALY percent for age groups over 50 was less than that of upper-middle-income countries, the USA, and the global average. However, the DALY percent of the 25–49 age group exceeded the global average from 2002 and exceeded that of the USA from 2017. The DALY percent of the 10–24 age group surpassed the global average in 2007 and surpassed that of the USA from 2008.

### The age-related trend of major neurological and mental disorders from 1990 to 2019

**FIGURE 3:**
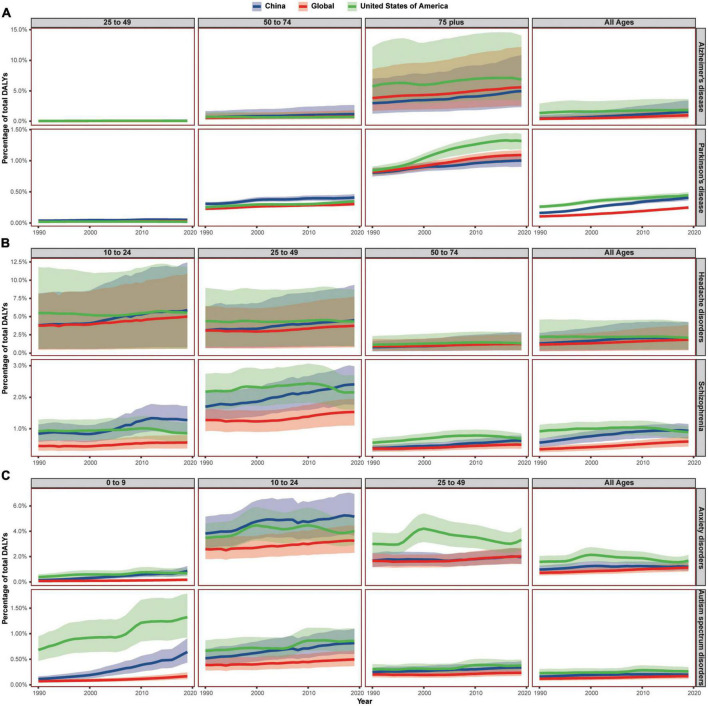
The burden trend of six common neurological and mental disorders in China, the United States of America, and the world, by age group. **(A)** Showed the DALY percentage of Alzheimer’s disease and other dementias and Parkinson’s disease; **(B)** showed the DALY percentage of headache disorders and schizophrenia; **(C)** showed the DALY percentage of anxiety disorders and autism spectrum disorders. Shaded sections indicate 95% uncertainty intervals. DALYs, disability-adjusted life-years.

**FIGURE 4:**
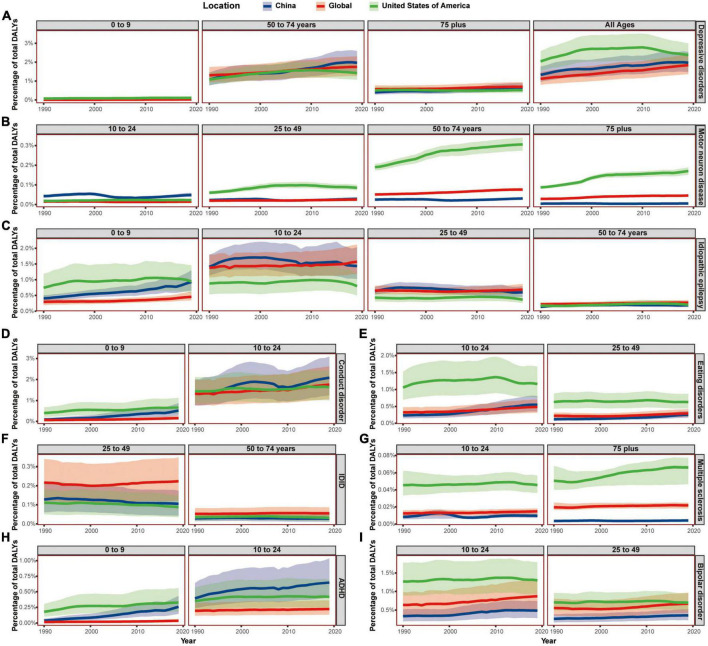
The burden of depressive disorders and other neurological and mental disorders, by age groups. **(A)** Showed the DALY percentage of depressive disorders; **(B)** showed the DALY percentage of motor neuron diseases; **(C)** showed the DALY percentage of idiopathic epilepsy; **(D–I)** showed the DALY percentage of conduct disorders, eating disorders, Idiopathic developmental intellectual disability, multiple sclerosis, attention-deficit/hyperactivity disorder, and bipolar disorders, respectively. Shaded sections indicate 95% uncertainty intervals. DALYs, disability-adjusted life-years; IDID, idiopathic developmental intellectual disability; ADHD, attention-deficit/hyperactivity disorder.

The all-ages Chinese DALY percent caused by Alzheimer’s disease and other dementias, Parkinson’s disease, headache disorders, anxiety disorders, autism spectrum disorders, depressive disorders, and conduct disorder lied between that of the USA and the world from 1990 to 2019, increasing similarly to neurological disorders or mental disorders. However, the DALY percent of China had exceeded that of the USA in some age groups (Figures 3, 4). Even the all-ages Chinese DALY percent caused by schizophrenia surpassed that of the USA in 2016.

For Alzheimer’s disease and other dementias, the Chinese DALY percent was lower than that of the USA and the world in the 75+ age group. In the 50–74 age group, the Chinese DALY percent was higher both than that of the world and the USA from 1990 and increasing steadily, while that of the USA was on a decrease. In the 25–49 age group, the Chinese DALY percent also showed the fastest increase and exceeded that of the USA in 2013.

For Parkinson’s disease, the DALY percent of China in the 75+ age group was lower than that of the USA and the world. However, the Chinese DALY percent was much higher than that of the USA and the world in both the 50–74 and 25–49 age groups. Both Alzheimer’s disease and Parkinson’s disease were neurodegeneration diseases with a later onset and evaluated from 25 years old in GBD 2019.

For headache disorders, the Chinese DALY percent of the 50–74 age group showed an upward trend and exceeded that of the USA in 2019, yet the DALY percent of the 75+ age group were lower than that of the USA and the world. The Chinese DALY percent of the 25–49 and 10–24 age groups exceeded that of the USA from 2017 and 2016, respectively. The 0–9 Chinese age group showed a similar increasing trend with the all-ages.

For schizophrenia, the Chinese DALY percent in the 75+ age group was lower than that of the USA but surpassed the global average in 2016. The Chinese DALY percent of the 50–74 age group was increasing while still higher than the global average and lower than that of the USA from 1990 to 2019. However, the Chinese DALY percent surpassed that of the USA from 2015 and 2005 in the 25–49 and 10–24 age groups, respectively. The evaluative starting age of DALYs caused by schizophrenia was 10.

For anxiety disorders, the Chinese DALY percent of people over 25 years old were lower than that of the USA, fluctuating around the global average. But in 10–24 age group, the Chinese DALY percent were higher than that of the USA and the world from 1990 to 2019. In 0–9 age group, the Chinese DALY percent were higher than the global average, rose rapidly, and surpassed that of the USA from 2018.

For autism spectrum disorders, the Chinese DALY percent were lower than that of the USA and the world in 75+ age group while higher than global average but lower than that of the USA in 25–49, 10–24, and 0–9 age groups. Then in 50–74 age group, the Chinese DALY percent exceeded that of the world from 1994.

For depressive disorders, the Chinese DALY percent of 10–24 and 25–49 age groups were lower than that of the USA and fluctuating around the global average. The Chinese DALY percent of 0–9 age group were lower than that of the USA but increasing and surpassed that of the world from 1993. The Chinese DALY percent of 75+ age group were lower than global average but increasing and exceeded that of the USA from 2006. In 50–74 age group, the Chinese DALY percent was the lowest at first but increasing rapidly and surpassed both the global average and that of the USA from 2007.

For conduct disorder, the Chinese DALY percent of the 10–24 age group were higher than the global average and increasing and exceeded that of the USA from 1997. The Chinese DALY percent of the 0–9 age group were lower than that of the USA and higher than global average and increasing. The evaluative age of conduct disorder in GBD 2019 was below 25.

For eating disorders, the Chinese DALY percent of the 25–49 age group was lower than that of the USA and the world, and the Chinese DALY percent of 10–24 age group was also lower than the other two in the first but increasing and surpassed the global average from 2009. The evaluative age of burdens of eating disorder was below 50. The eating disorder DALYs of 0–9 age group only accounted for a small part of the all-ages, and the Chinese DALY percent in this age group was higher than the global average and lower than that of the USA, but on a steady rise.

For motor neuron diseases, the DALY percent of the USA was the highest in 0–9 and 25–49, 50–74, and 75+ age groups. The DALY percent of China were the lowest in both 50–74 and 75+ age groups and were in the middle in 0–9 age groups. However, the Chinese DALY percent surpassed that of the world in the 25–49 age group and was even higher than that of the world and the USA in the 10–24 age group.

For idiopathic developmental intellectual disability, the DALY percent of China were the lowest in the 0–9, 50–74, and 75+ age groups and were lower than that of the world but higher than that of the USA in both the 10–24 and 25–49 age groups. The Chinese DALY percent showed a downward trend in most age groups except the 0–9 age group.

For multiple sclerosis, the DALY percent of China was the lowest while that of the USA was in the highest for people aged above 10 years. However, the gap was narrowing, and the ratio of Chinese DALY percent to DALY percent of the USA was increasing from 6.5% in the 75+ age group to 7.4% in the 50–74 age group to 12.4% in the 25–49 age group and to 20.6% in the 10–24 age group.

For idiopathic epilepsy in all-ages groups, the DALY percent of the USA was the lowest, the DALY percent of China was the highest in 1990 but reducing from 1998, and the DALY percent of the world was increasing slowly and surpassed that of China from 2006. The DALY percent in the 10–24 and 25–49 age groups showed a similar trend. However, in the 50–74 and 75+ age groups, the DALY percent of China were the lowest and that of the world were the highest. However, in the 0–9 age group, the DALY percent of the USA was in the top but in the middle in China but increasing rapidly.

For attention-deficit/hyperactivity disorder, the DALY percent of China was highest in most age groups except in the 0–9 age group, while that of the world was always lowest. Although the Chinese DALY percent showed a downward trend in all-ages groups, the percent was both increasing in the 10–24 and 0–9 age groups.

Although the DALY percent of burdens caused by motor neuron diseases, multiple sclerosis, and idiopathic epilepsy showed different trends with that of neurological disorders in all-ages group, they also showed age-related patterns and only accounted for 14.9% of the burdens caused by neurological disorders. Similarly, the DALY percent of burdens caused by attention-deficit/hyperactivity disorder and idiopathic developmental intellectual disability presented a disparate trend with that of mental disorders in the all-ages group, while they also showed age-related pattern and only accounted for 3.2% of the burdens caused by mental disorders. The only disorder that showed a totally different trend from other mental disorders was bipolar disorders. The Chinese DALY percent caused by bipolar disorders were lower than that of the world and the USA in all the age groups evaluated, while the burdens of bipolar disorders only accounted for 5.3% of the burdens caused by mental disorders.

### The age-related trend in body weight in the past half-century

**FIGURE 5:**
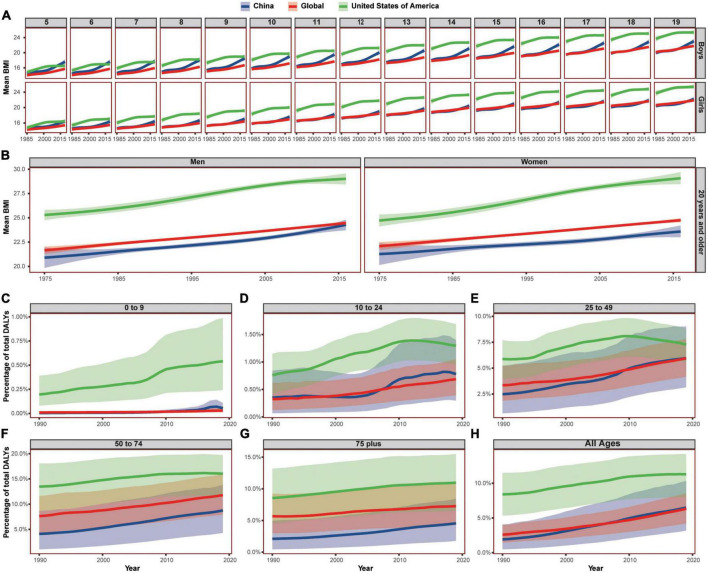
The trend of BMI and BMI-induced DALYs in China, the United States of America, and the world, by age and sex group. **(A)** Showed the BMI of adolescent aged from 5 to 19, **(B)** showed the BMI of adults over 20 years old. **(C–H)** Showed the DALYs induced by high BMI in different age groups. Shaded sections indicate 95% uncertainty intervals. BMI, body mass index; DALYs, disability-adjusted life-years.

Body weight also showed an age-related trend in the past decades. During the past 50 years, people are gaining weight globally (Figure 5). For adults over 20 years, the Chinese BMI was lower than the global average and that of the USA both in men and in women. However, the increasing pattern was different in children and adolescent (5–19 age group).

For boys, Chinese BMI exceeded the global average from 2005 in the 19-year age group, exceeded the global average from 1991 in 18 year age group, exceeded the global average from 1988 in the 17-year age group, exceeded the global average from 1986 in the 16-year age group, and exceed global average from 1985 in the 5–15 years age groups. The BMI of Chinese boys surpassed that of the USA from 2018 in the 7-year age group, surpassed that of the USA from 2016 in the 6 year age group, and surpassed that of the USA from 2012 in the 5-year age group.

For girls, Chinese BMI exceeded the global average from 2013 both in the 19- and 16-year age groups, exceeded the global average from 2015 in the 17–18 age groups, exceeded the global average from 2012 in the 15-year age group, exceeded the global average from 2010 in the 14-year age group, exceeded the global average from 2009 in the 12–13 age groups, exceeded the global average from 2008 in 11 age group, exceeded the global average from 2007 in 10 age group, exceeded the global average from 2005 in 9 age group, and exceeded the global average from 1985 in 5–8 age groups.

The DALY percent induced by high BMI (over 25) also showed a similar age-related trend. The all-ages Chinese percent was lower than that of the USA and the world at first but increased rapidly and surpassed the global average from 2007. The Chinese DALY percent exceeded that of the world from 2010 in 25–49 age group, exceeded that of the world from 2008 in 10–24 age group, and exceeded that of the world from 2009 in 0–9 age group. However, in age group over 50, the Chinese percent was lower than that of the world and the USA.

### The age-related trend in disease burdens induced by dietary risks from 1990 to 2019

Both diseases and body weight are closely associated with diet. Then, diet is among the most important factors regulating DALYs (48).

From 1990 to 2019, the all-ages DALY percent induced by dietary risks was decreasing in the USA while increasing in China, and the Chinese percent was higher than the world and surpassed that of the USA from 2000. The DALY percent induced by both risks of low in certain foods and high in certain foods showed the similar trend.

**FIGURE 6:**
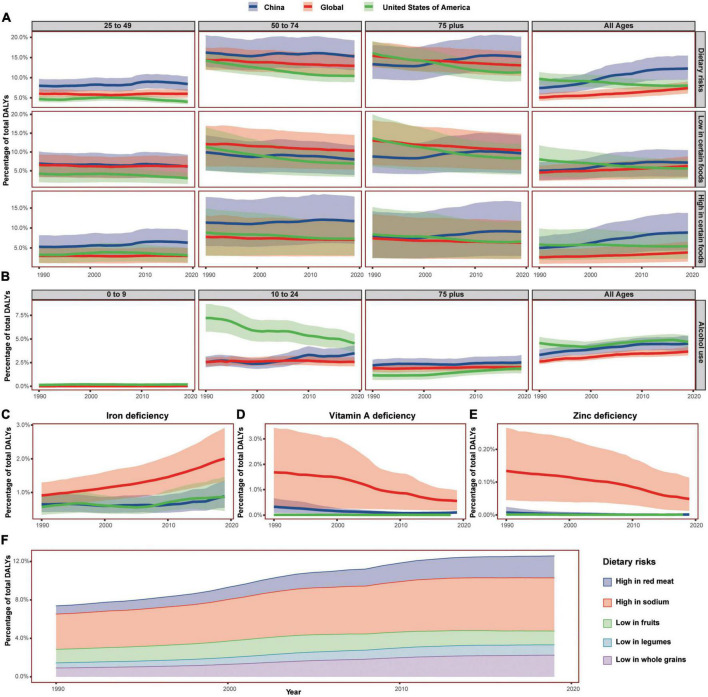
The DALYs induced by diet-related factors in China, the United States of America, and the world in 1990–2019. **(A)** Showed the DALYs induced by total dietary risks, diet high in certain foods, and diet low in certain foods by age group. **(B)** Showed the DALYs induced by alcohol use by age group. **(C–E)** Showed the DALYs of 0–9 age group induced by deficiencies of iron, vitamin A, and zine C, respectively. **(F)** Showed the trend of DALYs induced by top 5 dietary risks in China. Shaded sections indicate 95% uncertainty intervals. DALYs, disability-adjusted life-years.

The Chinese DALY percent induced by dietary risks in 75+ age group exceeded that of the USA from 2003 and exceeded the global average from 2004. The Chinese DALY percent induced by dietary risks in 25–49 and 50–74 age groups were both higher than that of the USA and the world. For the risks high in certain foods, the Chinese DALY percent were higher than global average, surpassed that of the USA from 1998 in all-ages group, surpassed that of the USA from 2001 in 75+ age group, and surpassed that of the USA from 1990 in 25–74 age groups. For the risks low in certain foods, the Chinese DALY percent exceeded that of the USA from 2004 in all-ages group, exceeded that of the USA from 2006 in 75+ age group, exceeded that of the USA from 2002 in 50–74 age group, and exceeded that of the USA from 1990 in 25–49 age group (Figure 6). The top five dietary risks in 2019 were high in sodium, low in whole grains, high in red meat, low in fruits, and low in legumes in China. Except low in fruits, the Chinese DALY percent induced by other four risks were increasing from 1990 to 2019.

During 1990–2019, the DALY percent induced by iron deficiency, vitamin A deficiency, and zinc deficiency in most age groups were all decreasing in China, the USA, and the world, while the DALY percent induced by iron deficiency showed an opposite but increasing trend in the 0–9 age group. Then in the 0–9 age group, the DALY percent induced by vitamin A deficiency showed a downward trend in both the USA and the world while showed first decreased and then increased trend in China. The DALY percent induced by zinc deficiency also presented a similar trend in 0–9 age groups.

The DALY percent induced by alcohol use also showed an upward trend in China, the USA, and the world in all-ages group, and the Chinese DALY percent was lay between that of the USA and the world. However, the Chinese DALY percent induced by alcohol use was lower than that of the USA and the world in the 25–49 age group, the Chinese DALY percent was lower than that of the USA but increasing rapidly in the 10–24 age group, and the Chinese percent of the 0–9 age group even surpassed that of the USA in 2019. The Chinese DALY percent induced by alcohol use was higher than that of the USA and the world in the 75+ age group, and the Chinese percent were lower than that of the USA in 2007 in the 50–74 age group.

### The diet composition changes in the past six decades

**FIGURE 7:**
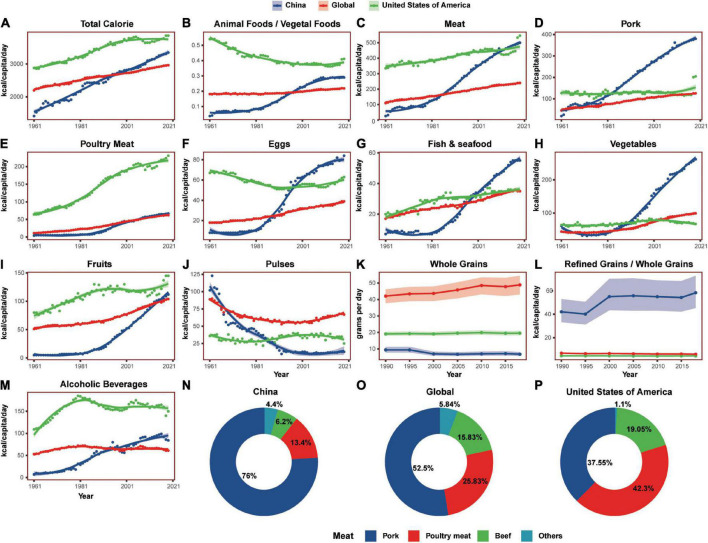
The diet composition differences between China, the United States of America, and the world during the past six decades. **(A,C–K,M)** Showed the daily consumption of total calorie, meat, pork, poultry meat, eggs, fish and seafood, vegetables, fruits, pulses, whole grains, and alcoholic beverages, respectively. **(B,L)** Showed the ratios of animal foods to vegetal foods and refined grains to whole grains, individually. **(N–P)** Showed the proportion of major meat intake. Shaded sections indicate 95% uncertainty intervals.

From 1961 to 2019, people were taking in more and more calories globally. The fastest growth rate was in China, which was quicker than that of the USA and the global average (Figure 7). The USA had the highest animal foods/vegetal foods ratio than China and the global average, but it was in a slowdown trend. During the same time, the ratio was increasing rapidly in China.

Over the past six decades, meat consumption presented a sharp increase in China, and the same trend appeared in the consumption of major animal foods such as pork, poultry meat, eggs, and fish and seafood. From 1961 to 2019, both the total meat consumption and the pork consumption increased by 18 times, the poultry meat consumption increased by 15 times, and egg consumption increased by 9 times. The most popular meat in China in 2019 was pork and poultry meat, accounting for 76 and 13.4% of the total meat consumption, while beef accounted only for 6.2%. Then, the top 3 most consumed meat in the USA were poultry meat (42.3%), pork (37.6%), and beef (19.1%).

During the same time, the consumption of some vegetal foods was also increasing rapidly in China, including vegetables and fruits, while the consumption of pulses was decreasing. From 1961 to 2019, Chinese fruit consumption increased 21 times and the vegetable consumption increased more than 3 times, while pulse consumption reduced to 87% and the ratio of meat to pulses increased sharply. From 1990 to 2018, the consumption of Chinese whole grains reduced to almost 28%, while that of the USA increased to 16%. Simultaneously, the Chinese refined grain/whole grain ratio rise was up to 10 times that of the USA.

In addition, Chinese alcoholic beverage consumption was also increasing sharply and increased 11 times from 1961 to 2019.

**FIGURE 8:**
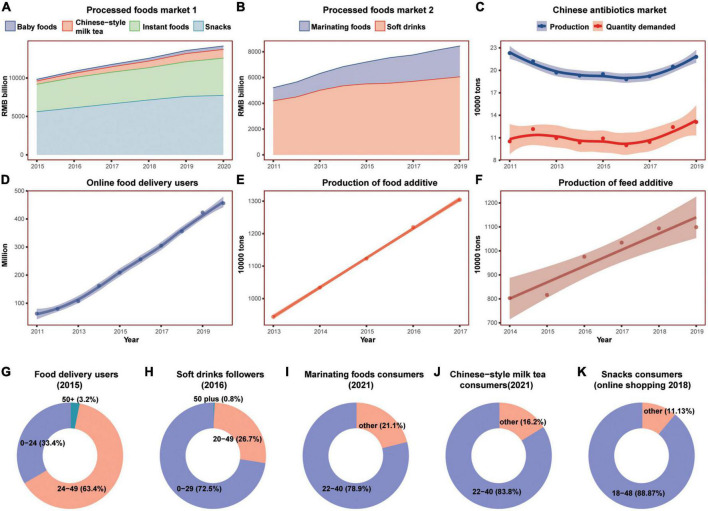
The surging consumption of processed foods in China in the past decade. **(A,B)** Showed the market expanding of six highly processed foods; **(E,F)** showed the rapidly increasing production of additives, and **(C)** showed the production and demand of antibiotics; **(D)** showed the rapidly increasing number of online food delivery users; **(G–K)** showed the age distribution of major processed food consumers. Shaded sections indicate 95% uncertainty intervals.

The last decade witnessed the expanding rapidly of Chinese highly processed food markets. As shown in Figure 8, accompanied by the surging demand in food additives, the market of Chinese marinating foods, snacks, instant foods, soft drinks, Chinese-style milk tea, and baby foods all presented increasing trend, demonstrating that the highly processed foods were replacing the fresh and low-processed foods in China. Another striking change in the Chinese diet was the surge of online food delivery. From 2011 to 2019, more and more Chinese people started eating takeaways. Semi-processed and high-processed takeout foods were replacing rapidly homemade foods.

It was worth noting that the consumers of highly processed foods and the user of food delivery were mainly people younger than 50. For soft drink followers, people below 29 accounted for 72.5% of the total. For Chinese-style milk tea and marinating foods, consumers of the 22–40 age group were 83.8 and 78.9% of the total, respectively. For snacks consumers shopping online, the 18–48 age groups made up of 88.9% of the total. For food delivery users, the 25–49 age group accounted for more than half (63.4%), and the people under the age of 25 accounted for 33.4%, while the people over 50 only accounted for 3.2%.

### The dramatic changes in food production over the past half-century

**FIGURE 9:**
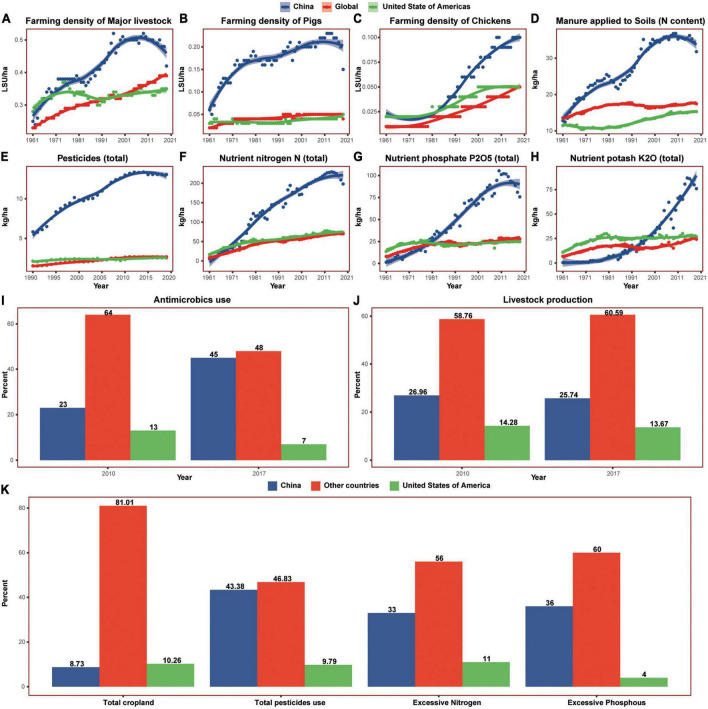
The changes of animal and vegetal foods production in China, the United States of America, and the world in the past six decades. **(A–C)** Showed the farming density of major livestock, pigs, and chickens, individually; **(D)** showed the manure applied to soils per hectare; **(E)** showed the total pesticides used for per hectare; **(F–H)** showed the usage of chemical fertilizer nitrogen, phosphate, and potash, respectively. **(I)** Showed the proportion of total antimicrobics usage in food animals and **(J)** showed the proportion of livestock production during the same time. **(K)** Showed the proportion of total cropland, pesticides use, excessive nitrogen and excessive phosphorus in 2014. Shaded sections indicate 95% uncertainty intervals, LSU, livestock unit; ha, hectare.

The past decades experienced dramatic changes not only in diet composition but also in food production in China. For animal food production, intensive livestock farming replaces traditional backyard production in the last decades, especially that of pigs and chickens (Figure 9). The intensive farming system requires premixed feedstuffs, antimicrobials, and other medicals to prevent animal diseases and stimulate their rapid growth. China has been among the largest production and consumption countries in antibiotics, additives, chemical fertilizers, and pesticides (79, 80, 83). The Chinese antimicrobial demand market and feed additives market were increasing rapidly in the last decade (Figures 8C,F). Contrary to the reduction in the USA, the antimicrobials used in food animals in China increased from 2010 to 2017, while the Chinese production of livestock decreased slightly during the same time. Following the increasing livestock numbers, the manure applied to the soil rose sharply in China compared to that of the USA and the world.

**FIGURE 10:**
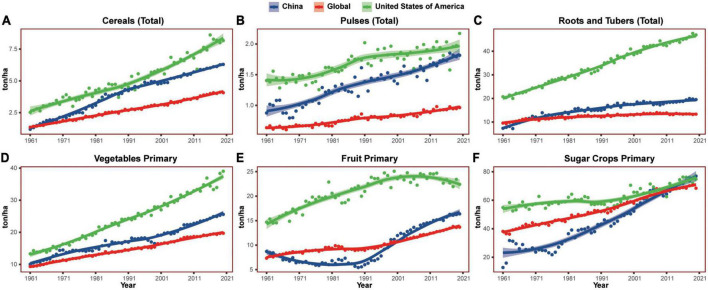
The major crops yield in China, the United States of America, and the world in the past six decades. **(A–F)** Showed yield per hectare of cereals, pulses, roots and tubers, vegetables, fruits, and sugar crops, respectively. ha, hectare.

Crop planting has also changed dramatically in the last decades. Most crop yields have been increasing in China (Figure 10), but both chemical fertilizer usage and pesticide usage are increasing more rapidly. From 1961 to 2019, chemical fertilizer usage (sum of nutrient nitrogen, phosphorus, and potash) in China increased 49 times, reaching almost 3 times that of the USA. From 1990 to 2019, pesticide usage doubled and reached 5 times that of the USA. Although China accounted for 8% of the world’s cropland in 2014, it used 43% of the world’s pesticides, resulting in 33% excessive nitrogen and 36% excessive phosphorus globally. However, the crop yields have not proportionally increased with the usage of fertilizer and pesticides, and the Chinese yields of major crops including cereals, pulses, vegetables, and fruits were all much lower than the USA.

### The expanding burden of neurological and mental disorders was associated with the great changes in diet composition and food production sustainability

**FIGURE 11:**
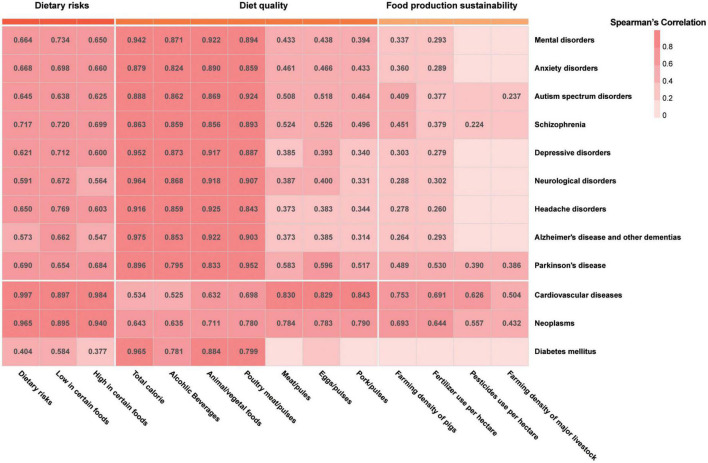
The correlation between disease burdens and diet quality and food production sustainability. Only the correlation coefficients with adjusted *p*-value of below 0.05 were shown in the figure.

The all-ages DALY percent of cardiovascular diseases, neoplasms, and diabetes mellitus was undoubtedly positively correlated with total dietary risk burdens because of the calculation method in GBD 2019 (Figure 11). The positive correlation between these disease burdens with low in certain foods and high in certain foods was also natural. It was worth noting that the burdens of the three dietary risks were significantly positively correlated with the all-ages DALY percent of major neurological disorders (headache disorders, Alzheimer’s disease and other dementias, and Parkinson’s disease) and mental disorders (anxiety disorders, autism spectrum disorders, schizophrenia, and depressive disorders).

Moreover, the correlation analysis also presented a positive correlation between the DALY percent of cardiovascular diseases, neoplasms, and diabetes mellitus with poor diet quality indicators including the consumption of total calorie and alcoholic beverages and ratios of animal foods to vegetal foods and poultry meat to pulses. The positive correlation between these poor diet quality indicators and the above neurological and mental disorders was even stronger. In addition, the ratios of meat to pulses, eggs to pulses, and pork to pulses were positively correlated with the DALY percent caused by cardiovascular diseases, neoplasms, Parkinson’s disease, headache disorders, depressive disorders, anxiety disorders, schizophrenia, and autism spectrum disorders.

More notably, two food production sustainability indicators, the farming density of pigs and the chemical fertilizer (N + P + K) usage per hectare, are significantly positively correlated with the DALY percent caused by the above disease except diabetes. In addition, the farming density of major livestock is also positively correlated with the DALY percent of cardiovascular diseases, neoplasms, Parkinson’s disease, and autism spectrum disorders. The total pesticide usage per hectare significantly positively correlated with the DALY percent of cardiovascular diseases, neoplasms, Parkinson’s disease, and schizophrenia.

## Discussion

### Main findings

China has been experiencing the industrialization of food production over the past six decades. Compared with the USA and the world, China has used much more fertilizer and pesticides in crop planting. The Chinese farming density of major livestock such as pigs and chickens is also much higher, accompanied by the overuse of antimicrobics. The livestock manure applied to soils per hectare is much more. All these suggest the reduction of food production sustainability in China.

During the same time, the Chinese diet quality has been reduced remarkably. The consumption of total calorie and alcoholic beverages are increasing rapidly. The Chinese ratio of animal foods to vegetal foods has been increasing since 1961 while that of the USA has shown a downward trend. The major animal foods consumed in China are pigs and poultry, which are among the livestock being feed with the most amount of antimicrobics (77, 79). Although the consumption of vegetables and fruits is also increasing, the consumption of pulses and whole grains is reducing. The Chinese ratio of meat to pulses has exceeded that in the USA since 1991 and the Chinese ratio of refined grains to whole grains is also much higher than that of the USA. Furthermore, the consumption of processed foods has been increasing sharply in the last decade, and homemade foods are being replaced by processed foods, delivery foods, and restaurant foods. All the above indicate that China is going through the westernization or industrialization of diet.

Moreover, the consumers of processed foods in China were mostly young people. The BMI trend shows that Chinese children and adolescents were gaining weight quicker than those from the USA and the world, with the younger the quicker, although the BMI of Chinese adults was lower than that of the USA and the world. The Chinese DALYs induced by high BMI also showed an age-related pattern. The DALYs induced by high BMI were increasing rapidly in people below age 50, especially the young under age 25 in the last decade.

The age-related diet quality reduction was accompanied by the increasing trend of disease burden induced by dietary risk factors. The Chinese DALY percent induced by dietary risks were growing and surpassed that of the USA from 2000 in all-ages group, from 2003 in the 75+ age group, and from 1990 in the 25–74 age group. This age-related pattern in China appeared more apparent and earlier in risks of a diet high in certain foods than low in certain foods. The global dietary risk changes had been analyzed by the reports of GBD 2017 (48). and GBD2019 (50). Since the current evaluations of disease burden attributed by dietary risks were mostly assessed the later-onset diseases such as cardiovascular diseases, neoplasms, and diabetes mellitus from people over 25 years old, and it took time for the health results of poor diet in young people to emerge, the Chinese DALYs induced by dietary risks were probably underestimated. In addition, although the disease burden induced by nutritional deficiencies reduces in the past 30 years, the Chinese DALYs induced by vitamin A deficiency and zinc deficiency have started to increase again in the 0–9 age group in the last decade. Besides, the Chinese DALYs induced by alcohol use are also increasing remarkably in the 10–24 age group, opposing the downward trend in the USA and the world, and the Chinese DALYs are also increasing rapidly in people below 10 years of age.

Following the reduction in diet quality and food production sustainability, the Chinese trend of neurological disorders, mental disorders, and major chronic diseases presented an age-related increased pattern. The disease burdens of China were usually less in older people than that of the USA and the world, while climbed skyward and surpassed the global average or/and that of the USA in the younger one in the last decades. The Chinese burden of cardiovascular diseases and depressive disorders in the 75+ age group surpassed that of the USA from 1997 and 2006, individually. The Chinese burden of Alzheimer’s disease and other dementias, Parkinson’s disease, neoplasms, and mental disorders in the 50–74 age group surpassed that of the USA from 1990, 1990, 2004, and 2017, respectively. The Chinese burden of schizophrenia, headache disorders, idiopathic epilepsy, and diabetes mellitus in the 25–49 age group surpassed that of the USA in 2015, 2017, 1990, and 2017, respectively. The Chinese burden of anxiety disorders, conduct disorders, motor neuron disease, and neurological disorders in the 10–24 age group surpassed that of the USA in 1990, 1997, 1990, and 2003, respectively. By the way, three disorders were presenting an age-related upward trend although their disease burdens were lower than that of the USA. The Chinese burden caused by autism spectrum disorders exceeded the global average in the 50–74 age group from 1993, the Chinese burden caused by idiopathic developmental intellectual disability exceeded the global average in the 25–49 age group from 1990, and the Chinese burden caused by eating disorders exceeded the global average in 10–24 age group from 2009.

The burden changes of the above diseases are associated with the dietary transition. Not only the burdens of cardiovascular diseases and neoplasms but also the burdens of the above neurological and mental disorders were all increasing with dietary quality reduction. The poor diet quality indicators included higher ratios of animal foods to vegetal foods, poultry meat to pulses, meat to pluses, eggs to pulses, and pork to pulses, higher calorie intake, and higher alcohol intake. Furthermore, the burdens of cardiovascular diseases, neoplasms, and Parkinson’s disease were all increasing with the increase in fertilizer use, pesticide use, the farming density of pigs, and farming density of major livestock. Moreover, the burdens of mental disorders, anxiety disorders, autism spectrum disorders, schizophrenia, depressive disorders, neurological disorders, headache disorders, and Alzheimer’s disease and other dementias were all positively correlated with two poor food production sustainability indicators including the farming density of pigs and fertilizer use per hectare. The burdens of schizophrenia were also positively correlated with the pesticide usage per hectare, and the burdens of autism spectrum disorders were also positively correlated with the farming density of major livestock.

### The industrialization of gut microbiota and increase of diseases susceptibility following the industrialized diet

The association between the burdens of neurological and mental disorders with dietary transition is not unreasonable, and the industrialization or westernization of gut microbiota could be the key link (6, 9, 29, 30).

The rapid dietary transition and urbanization are driving the industrialization or westernization of gut microbiota (52, 53, 84). In the USA, immigrants westernized their gut microbiota after migration, and the western diet played a great role in this transition (51, 85). In China, urbanization and dietary transition changed human gut microbiota (86, 87), even the herdsmen in remote Tibet were experiencing the gut microbiota transition (88). Poor diet quality that occurs in the dietary transition including high calorie, high alcohol consumption, high refined grains while low whole grains, high-processed foods, high meat while low pulses, and a variety of additives, similar to the western diet, could disturb gut microbiota, impair brain function through microbiota—gut–brain axis, and increase the susceptibility of many neurological and mental disorders (6, 8, 9, 29, 30, 35, 41, 89, 90).

Residues of pesticides, heavy metals, and antibiotics in foods are also important diet-related factors interfering with gut microbiota and brain function (43, 91–96). Tremendous use of fertilizers and pesticides have been used in China’s agriculture in the past decades, reducing the diversity of soil microbiota and soil fertility (97–99), leaving high and probably growing persistent organic pollutions (POPs) and heavy metals in soil and water (100, 101). Many POPs such as organochlorine and heavy metals such as cadmium and lead are carcinogenic and neurotoxic. Crops and other plants absorb these toxic substances from the environment as they grow. Then, livestock and fish accumulate more toxic substances in the body from these grains, grass, and environment. These toxic substances will probably pass to humans through food chains, disrupt human gut microbiota, and induce neurotoxicity and brain impairment through microbiota–gut–brain axis (43, 92, 94, 102–104).

The impact of antimicrobics originating from food animals is more complicated. Antibiotics or antimicrobics have been widely used in industrialized food animal production including pigs, poultry, cattle, and aquiculture for growth promotion, disease prevention, and therapy (105). Pigs are top 1 among the animals fed most antimicrobics (77, 79), and pork is more consumed in China than in the USA. It is not surprising that higher burdens of neurological and mental disorders followed a higher ratio of pork to pulses and farming density of pigs. The farming method disrupts animal gut microbiota [kill sensitive bacteria and prompt the thriving of antimicrobial resistance (AMR) bacteria] and reduces α-diversity, produces animals with a big and fat body but compromised immunity, creates pollution in the environment including AMR genes, heavy metals, antimicrobics and other drugs, greenhouse gases, and unintentionally but objectively avails the evolution of pathogens (106, 107). On the other hand, the antimicrobial residues and AMR genes in animal foods will transmit to humans through the food chain (108, 109), disturb gut microbiota and decrease α-diversity, induce overweight and obesity (110, 111), and impair brain function (6, 95, 96).

Long-term fertilization, whether chemical fertilizer or manure fertilizer or blended fertilizer, would increase the heavy metals including cadmium and arsenic in the soil (112, 113). Furthermore, a substantial portion of the antimicrobics, feed additives, and AMR genes excreted by animals also disrupted soil microbiota and returned to food chains by applying manure to the soil. These harmful substances were absorbed by crops and other plants from the soil and water and then transferred to food animals or humans (106, 114), impairing body and brain health finally (95, 111). Thus, animal foods often contain more harmful substances than vegetal foods following the biological huge concentration of pesticides, heavy metals, and antimicrobics and the transmission of AMR genes. It is not surprising that higher ratios of animal foods to vegetal foods, major meat to pulses, and eggs to pulses were accompanied by higher disease burdens of neurological and mental disorders.

In contrast to the increase of the above harmful substances, the nutrient density decreased in the foods. The reduction of nutrition value would further exacerbate the poor diet problem (115). Although there is limited research that directly analyses the influence of crop nutrition due to modern agriculture, several research studies showed that fertilizer, pesticides, heavy metals, pollutants, global warming, and antibiotics changed soil pH value, disturbed soil ecosystem, and plant root microbiota (98, 99, 116–118). Similar to the animal gut microbiota, the root microbiota not only play an important role in plant growth and health but also influence crop performance (119–122). Soil microbiota diversity especially of bacteria declined (123) and root microbiota changed (117) following the increase of atmospheric CO2 and temperature, and nutrient density reduced in a range of staple crops including wheat, rice, barley, field peas, soybeans, chickpea, potatoes, fruits, C3 vegetables during the same time (124, 125). The decreased nutrition was protein, zinc, and iron, among the most common nutritional deficiencies (115, 125). Repairing the soil ecosystem and root microbiota through applicating beneficial microbiota could not only promote crop growth, immunity, and nutrient utilization but also improve nutrition value and reduce the usage of pesticides and fertilizer (121, 122). Inoculating *Rhodopseudomonas palustris* strain PS3 not merely significantly improved the yield of marketable tomato fruit but also promoted the sweetness, taste, and the content of vitamin C, total phenolic compounds, and lycopen (126). Applicating Azospirillum along with half a dose of nitrogen and phosphate fertilizers not only increased the yield of sesame but also enhanced the oil quality with lower acid value and healthier fatty acid composition (127). Inoculation with *Azospirillum brasilense* increased the yield of hydroponic lettuce and increased the leaf accumulation of important minerals such as iron and zinc (128). Our study in crops also supported the use of Lactobacillus fermented products as a safe alternative to pesticides. In addition, healthy soil microbiota could possibly decrease the harmful substances absorption of crops. Antibiotic exposure not only reduced soil bacteria diversity but also increased AMR gene accumulation in vegetables (118), while indigenous soil microbiota played a great role in preventing the spread of AMR genes (129).

The nutrients in the meat are closely related to the gut microbiota of the animal. Antimicrobics used in the farming process could disrupt the gut microbiota of animals, promote body weight gain, also facilitate fat deposition, and suppress muscle development (130, 131), making similar influences with what it does in the human body (96, 110, 132, 133). Compared to wild fish with undisturbed gut microbiota, cultured fish had less omega-3 PUFA, more omega-6 PUFA, and more saturated fatty acids (134, 135). Compared to traditional grass-fed beef with undisturbed gut microbiota, modern grain-fed beef had more saturated fatty acids and less omega-3 PUFA, conjugated linoleic acid, β-carotene, α-tocopherol, vitamin B12, iron, zinc, and glutathione (136–138). Researchers have been seeking more sustainable alternatives to antibiotics for decades, and probiotics and prebiotics could be a suitable one (139). Probiotics or/and prebiotic supplementation enhanced the immunity of the animal, increased feed digestibility, promoted the growth and reproductive performance, improved the yield and quality of meat, milk, and eggs, increased the content of free amino acids, eicosapentaenoic acid (EPA), docosahexaenoic acid (DHA), superoxide dismutase, glutathione peroxidase, decreased the content of saturated fatty acids, triglyceride, and cholesterol (140–146), and reduced the noxious gas emission including ammonia and sulfuretted hydrogen (147) and harmful substance accumulation such as chromium (146). Our study in food animals also found that feeding certain Lactobacillus strains fermented feed not only increased feed conversion ratio, decreased noxious gas in feces, and reduced aggressive behaviors, but also lesson the cholesterol in eggs and meat and increased the lean body mass and content of EPA and DHA.

All the above changes both in dietary structure and in food production sustainability were pushing the industrialization of human gut microbiota. Current gut microbiota research has focused more on bacteria, although it is still controversial, and a growing body of research found that industrialization decreased the α-diversity but increased the β-diversity of human gut microbiota, decreased the fiber-degrading genes, and increased complex chemicals-degrading genes (51, 53, 85, 87, 148, 149). Meanwhile, overweight and obesity people also owned gut microbiota with lower α-diversity compared to normal bodyweight control, especially the people eating more high-fat red meat and having higher veterinary antibiotic levels in their body (111, 150–152). Many studies showed that the patients with mental and neurological disorders including depressive disorders, schizophrenia, Alzheimer’s disease, Parkinson’s disease, autism spectrum disorders, attention-deficit/hyperactivity disorder, and eating disorders also had a lower α-diversity in gut microbiota compared to healthy controls (16, 153–157). The patients with many chronic diseases such as cardiovascular diseases, cancers, and diabetes mellitus presented a lower α-diversity in gut microbiota, too (158–162). The abnormal gut microbiota could impair brain function through microbiota–gut–brain axis, making the host more susceptible to the above diseases (6–9, 163). Furthermore, the industrialized gut microbiota shaped by a poor diet containing unsafe substances are the compromised gut microbiota, reduce the host’s ability to excrete heavy metals and harmful substances, and increase the susceptibility to various diseases including neurological and mental disorders (6, 29, 30, 36, 41, 164, 165). Finally, it would become a vicious cycle without dietary quality and food production sustainability improvement.

Yet, the association between cancer, cardiovascular diseases, neurological disorders, and mental disorders with food production sustainability was more insidious. It is well known that environment POPs, pesticides, heavy metal, and some antimicrobics would increase the risks of cancer and cardiovascular diseases (166, 167). Among the neurological and mental disorders, neurodegenerative diseases such as Parkinson’s disease and neurodevelopmental disorders such as autism spectrum disorders are more sensitive to the above harmful residues in foods (92–94, 104, 168). It is not surprising that the disease burden of Parkinson’s disease is positively correlated with the usage of pesticides and fertilizers and the farming density of major livestock and pigs. The association between these harmful residues and schizophrenia was recently noticed (43), and their links with other neurological and mental disorders are probably going to be found in the future. Food production sustainability not only affects the resilience to a possible full-blown AMR crisis or climate changes in the future (107, 169) but also influences the body and brain health of current people and future generations.

### The differences in diet composition and food production sustainability between China and the USA

The Chinese food system has been increasingly industrialized in the past decades, and despite the growing similarities with the USA, their differences are still stark.

#### Differences in diet composition

Processed foods already make up for more than 60% of the current USA diet (62). Its proportion in China is increasing rapidly, almost 30% in total and nearby 50% for children and adolescents (58, 61). There are more differences in processed foods between the USA and China.

The FEDERAL court of the USA approved The Nutrition Labeling and Education Act (NLEA) (Public Law 101-535) in 1990 not only regulated the nutrition labeling of packed foods in detail, but also emphasized related education for consumers to choose healthy foods. In 2014, the US Food and Drug Administration (FDA) issued Food Labeling: Nutrition Labeling of Standard Menu Items in Restaurants and Similar Retail Food, asking chain restaurant (more than 20 branch chain) and retail food producer indicate the food that contains energy, retail food firms and vending machines also need the food that contains energy labeling. In 2016, FDA updated the Nutrition Facts Label, requiring more specified description of nutrient labeling and health effects for consumer to do healthier choice.

Chinese Ministry of Health has required packaged foods to be labeled with nutrition facts since 2002 like the NLEA 1990 of the USA but has not proceeded with sufficient public education. According to the 2020 reports of the United Nations International Children’s Emergency Fund, processed foods whose nutritional composition does not meet World Health Organization recommendations including soft drinks, snacks, baby foods, and fast foods have been increasingly popular in Chinese children and adolescents in the last decade. These trends were in accordance with the obvious increase of burdens induced by high BMI in the young. More strict marketing restrictions, more taxes on highly processed foods, more clear nutrition labels such as front-of-pack labeling, and more understanding about healthier foods are all in urgent need to stop the worrying trend (170).

In addition, more education, research, and popular science on healthy eating are also needed very much in China since lots of people still choose food according to the traditional experiences and advertisement and marketing of foods, while the Chinese traditional food culture sometimes makes a negative impact in this respect. For example, the “the more refined grains and meat, the better” by Confucius encourages many to pursue finely processed and delicately cooked foods. The concept of “Alcohol is the quintessence of grains” and the Alcohol Culture (JiuWenHua) enforcing people drinking make most Chinese people unaware of the harm of alcohol.

#### Differences in food production sustainability

Although the problem of AMR in food animal production has been recognized worldwide, the policy in different countries is completely different. Antimicrobics in food animal production are now strictly prohibited in European Union but not completely prohibited in the USA, while the attitude of China is more compromised and reliable access to cheap meat seems to be more valued. Even so, the USA has banned the use of antibiotics as growth promoters and managed to stall decades of increasing antibiotic use and establish surveillance systems with which to hold policymakers accountable to stewardship commitments (105). While China has issued a series of policies to regulate antimicrobial use in the last decades and started to prohibit the use of drugs other than traditional Chinese medicine as feed additives since 1 July 2020 (Announcement of the Ministry of Agriculture and Rural People’s Republic of China No. 194.), the increasing trend does not seem not to be stopped (77, 79). Previous research found that China had a serious problem with the misuse and overuse of antimicrobics in food animal farming including the preference for new and highly effective drugs, the excessive use of non-prohibited and prohibited antibiotics, long-term and continuous use, the absence of veterinarians’ instructions, use of counterfeited and substandard products, repeated use of different brands but same substance, violating drug withdrawal time, etc (171–173). Although the percent of intensive livestock farming was increasing and up to 58% in 2017 in China (China Animal Husbandry and Veterinary Yearbook 2018), the majority were small-scale farms with insufficient pollution treatment conditions, making the monitor and control extremely difficult (106). Thus, more technical assistance for farmers on antimicrobial use, more regulations, and supervision are needed to stop the alarming increasing trend in antimicrobial use in food animal production.

For pesticides, lots of countries make clear regulations on the production, sale, and consumption of pesticides, and they also set maximum residue limits (MRLs, known as pesticide tolerances in the USA) to improve food safety. The USA has established a relatively complete legal system and relatively comprehensive MRLs for pesticide use, which are being updated as needed. In addition, the USA has proceeded with several long-term and systematic regulatory monitoring programs on pesticide residues. FDA has published annual reporters summarizing the results of the pesticide residue monitoring program since 1988 and conducted the Total Diet Study, which monitors pesticide residues in foods prepared as home cooking. The United States Department of Agriculture updated the database continuously according to its Pesticide Data Program results, too (174). Compared with the USA, China is a late starter in pesticide management. China issued the Regulations on Pesticide Administration in 1997 and constantly improved the related regulations, and its numbers of MRLs have been increasing rapidly since then. However, the biggest difference seemed probable not in regulations and MRLs numbers, but in supervision. It was reported that the detection rate of highly hazardous or banned pesticides in Chinese food was gradually decreasing, and the rate of violative residue was also reducing (83, 175, 176). However, there is still a gap between the violation rate and that of the USA, and it is difficult for China’s consumers to see detailed official reports on MRLs in food.

For fertilizer, less than half of it is utilized by crops globally, and the rest runs off farms into the natural environment and causes pollution and inducing ecosystem imbalances and biodiversity reduction. The nitrogen use efficiency (the ratio of nitrogen in harvested products) is only 0.25 in China compared to 0.68 in the USA and 0.42 in the world (169). While long-term excessive chemical fertilization will not only disturb soil microbiota and reduce soil fertility but also cause heavy metals pollution and other environmental pollution. However, it is possible to reduce chemical fertilizer use without decreasing crop yields (126, 177). A large-sale study proceeded in China reduced nitrogen fertilizer use by around one-sixth and increased crop yields by around 11%, and the results were mainly achieved by educating and training farmers on good management practices (178). China has initiated restrictions on heavy metal maximum limits in kinds of fertilizers, but only effective monitoring could stop this pollution from increasing.

### Future direction

Increasing research has indicated that mental disorders and neurological disorders are the diseases closely related to the dysbiosis of gut microbiota and dysfunction of microbiota–gut–brain axis in the past two decades, and improving that gut microbiota through diet quality enhancement is one of the best ways to prevent and alleviate the two brain disorders (6–11, 179–184). Although mounting studies showed the relationship between mental and neurological disorders with diet quality (27–35, 42, 44–47, 163), the relationship between the disorders and food production sustainability indicators is not well studied. This is the first research that analyzed the association between the burdens of mental and neurological diseases and dietary-related factors at such a macrolevel (country level).

Following globalization, diet composition and food production transition toward industrialization have become an almost irreversible trend. This tendency started in high-income countries, spread to the upper-middle income countries, and then accelerated in low-income and middle-income countries (55, 56, 58, 59, 79, 80, 105), followed by the surging of neurological and mental disorders burdens. China has made amazing economic development and successfully solved the problem of insufficient food in the last decades, but it also experienced increasing burdens of mental disorders, neurological disorders, cardiovascular diseases, neoplasms, and diabetes mellitus, following the reduction in dietary quality and food production sustainability. It is time to stop the rise of disease burdens and make more balanced progression.

Furthermore, development and disease burden are not an either-or choice. Albeit the USA is not the best in enhancing dietary quality and food production sustainability (48, 105), it has stopped and inversed some upward tendencies in disease burdens through the food system improvement. Dietary quality and food production sustainability might play a key role in the prevention of the above disorders. Thus, serious attention should be paid to promoting diet quality and food production sustainability; otherwise, the burden for neurological and mental disorders could expand rapidly in the next decade and the control could be more costly as we know those are the world’s most expensive diseases. Active prevention is usually a more cost-effective way than treatment afterward.

Although the industrialized food system feed a larger population than ever before, it also brings some problems. How to avoid the adverse effects including expanding of disease burden during developing should be the concern of every country. The experiences and lessons during the development of China and the USA not only help to improve themselves but also provide reference to the world. Every country should focus on diet quality and food production sustainability in diet industrialization, especially for low-income and middle-income countries.

For example, India has become the largest country of population development in the world, and the present comparative study between China and the USA has a very important reminder of India’s rapid rise. The present study argues that food processing and food security are the key to a country’s health and sustainable development, and without this key, human health and world peace and stability are difficult to maintain.

The COVID-19 pandemic has brought more challenge in disease burdens including neurological and mental disorders (70, 71). The pandemic probably induces diet quality reduction (72, 73, 185) while poorer diet quality was accompanied with higher risks and severity of COVID-19 infection (186, 187). In addition, pandemic increased the difficulty to access traditional treatments requiring in-person social interaction, resulting more and more patients without sufficient help and treatment (188). Thus, it is in urgent need for an effective remote intervention like diet quality improvement to prevent and alleviate neurological and mental disorders. This is not only a personal problem or a researcher’ subject, but also a topic should be concerned by all the world including policy maker, food producer, doctors and other health practitioner, researchers, and food consumers.

### Limitations

In the present study, we mainly focused on the association between total calories, alcohol beverages, animal foods to vegetal foods, and various meat to pulses with disease burden, the other diet quality indicators, including the ratios of refined grains to whole grains, processed foods to total dietary calorie, and omega-3 to omega-6 PUFA were not deeply analyzed. This is mainly due to their data being partially inadequate or unreliable but not because they were unimportant. Similarly, the association of antimicrobial use in food animal production was also not analyzed in depth due to insufficient data. Although we studied the differences in the disease burdens across age groups, no age-related correlation analysis was conducted due to data insufficiency. In the correlation analysis, the effects of sex, living environment, antibiotics use, and economic condition, on disease burden and diet were not analyzed because of the data inadequacies. More research is needed to figure out the impact of these factors on the association in the future.

## Conclusion

China has experienced a great increase in burdens of neurological and mental disorders in the past 30 years of rapid development process, although the corresponding DALY percent of the elderly is lower than the global average and the USA, the corresponding DALY percent of the younger generations is increasing faster than the global average and the USA. This age-related changing pattern in disease burden is greatly associated with the dietary transition during the past six decades. Following the reduction of diet quality characterized by high consumption of calories, alcohol, and processed foods, high ratios of animal to vegetal foods, and high ratios of major meat to pulses, the burdens of major mental disorders and major neurological disorders, cardiovascular diseases, neoplasms, and diabetes mellitus are increasing. Furthermore, the burdens of Parkinson’s disease, schizophrenia, autism spectrum disorders, cardiovascular diseases, and neoplasms are all associated with food production sustainability reduction. What is worse, children and teenagers are probably harmed more by this dietary transition than older people because they are more attracted to industrialized diet. However, it is inspiring that the burdens of the above diseases trend to reduce following the improvement in diet quality and food production sustainability in the USA. The right diet and sustainable food production would stop and/or even reverse neurological and mental disease burdens expanding.

## Data availability statement

The original contributions presented in this study are included in this article/supplementary material, further inquiries can be directed to the corresponding authors.

## Ethics statement

Ethical review and approval was not required for the study on human participants in accordance with the local legislation and institutional requirements. Written informed consent for participation was not required for this study in accordance with the national legislation and the institutional requirements.

## Author contributions

SL designed the study, collected and analyzed the data, draw the figures, and wrote the manuscript. FJ supervised the study and edited the manuscript. LW gave valuable suggestions. XW, XH, and TW offered help in data collection. All authors have contributed to the work and read and approved the final manuscript.
